# Problem Behaviors among Israeli Undergraduate Students: Applying Jessor’s Problem Behavior Theory among Young Adult Students

**DOI:** 10.3389/fpubh.2014.00273

**Published:** 2014-12-22

**Authors:** Liat Korn, Yael Shaked, Haya Fogel-Grinvald

**Affiliations:** ^1^Department of Health Management, School of Health Sciences, Ariel University, Ariel, Israel; ^2^Department of Mathematics, Faculty of Exact Sciences, Bar-Ilan University, Ariel, Israel

**Keywords:** problem behaviors theory, multiple problem behavior index, protective factors, risk factors, moderating effects, Israel, undergraduate students, health risk behaviors

## Abstract

**Purpose:** The current study tested the applicability of Jessor’s problem behavior theory (PBT) in Ariel University.

**Methods:** A structured, self-reported, anonymous questionnaire was administered to undergraduate students. The final study sample included 1,360 participants (882 females and 478 males, mean age 25, SD = 2.9, range = 17).

**Results:** Findings indicated that the PBT was replicated in this sample. As shown from the hierarchal linear regression model, religiosity and high-academic achievements were found to be strong and significant protective factors that reduce risk behaviors. Among young and religious students, the personal vulnerability has almost no impact on involvement in risk behaviors.

**Conclusion:** The PBT finds empirical support in this young adult undergraduate Israeli sample.

## Introduction

Problem behaviors refer to behaviors that transgress societal norms and can compromise health and development (1). Research on problem behaviors usually refers to adolescents in their different phases of development (2, 3). In this study, we explore the applicability of the problem behavior theory (PBT) as an explanation for problem behaviors among young adult undergraduate university students in Israel.

The PBT explains that behaviors such as delinquency, tobacco use, alcohol abuse, other illicit drug use, early sexual intercourse, aggression, and risky driving, are a product of integration between risk factors and the moderating or buffering effects of protection factors that may have an impact on exposure to risk. Risk factors raise the probability of involvement in risk behaviors by providing a model for problematic behavior, broadening possibilities of involvement in risk behaviors, and magnifying the personal vulnerability of problem behaviors. Protective factors reduce the probability of involvement in risk behaviors by providing a model of positive social behaviors, by means of social and personal supervision and control as well as a supportive social environment (4). Protective factors also refer to individual’s resilience – the maintenance of positive adaptation by individuals despite experiences of significant adversity (5). Both risk and protective factors are present in all of our social and personal systems (4, 6). According to this theory, health risk behaviors are related to each other and have a clustering effect. Additionally, in life situations faced by adolescents, the greater the risk factors are and the lesser the protective factors are, the greater the likelihood of the adolescent’s involvement in problem behaviors [Ref. (4) in: Ref. (1)].

Ndugwa et al. had already mentioned that in a large number of studies, psychosocial risk and protective factors have been shown to account for substantial amounts of variation in adolescent problem behavior for both male and female adolescents, for younger and older adolescents, and across groups varying in socioeconomic status, race, and ethnicity (1, 4, 7–9). A cross-national study conducted in both China and the United States on students in grades 7, 8, and 9, showed that despite mean differences in psychosocial protective and risk factors, as well as in the problem behaviors indicated, differences that may reflect societal variation, the explanatory model has, to a large extent, cross-national generality (6).

Even among college students (10), key predictors for smoking include controls protection, models risk, vulnerability risk, behavioral protection, and behavioral risk. Findings from a study conducted in both Georgia and Switzerland showed that the PBT finds empirical support in these Eurasian and Western European samples, and thus, Jessor’s theory holds value and promise for understanding the etiology of adolescent problem behaviors outside of the United States as well (11). In another study, factors affecting the transition from experimental smoking, at baseline, to two types of daily smoking: temporary daily smoking and continued daily smoking at a 1-year follow-up were examined. Important PBT-related predictors found for smoking were the number of friends who smoke, academic performance, as well as alcohol, marijuana, and other illicit drug use (12). Ndugwa et al. (1) claimed that measures of the theoretical psychosocial protective and risk factor concepts provided a substantial, multivariate, and explanatory account of adolescent problem behavior variation and demonstrated that protective factors can also moderate the impact of exposure to risk of poor urban adolescents in sub-Saharan Africa. More support for the PBT was provided by Lombe et al., who studied African American youth with respect to adolescent alcohol use. They found that depressive effects, delinquent behaviors and affiliation with delinquent peers were all found to be related to adolescent alcohol use (13).

In Israel, health risk behaviors have been previously examined as part of the HBSC study (health behavior in school-aged children) among a large representative sample of 6th, 8th, and 10th grade adolescents studying in the Jewish and Arab state including secular and religious school systems (14, 15). Various health risk behaviors such as water-pipe smoking were examined (16, 17). Findings showed that the association of water-pipe use with cigarette smoking, problem drinking and drug use is very strong. This suggests that despite the common lay perception among the Israeli public, that water-pipe use is only a social activity, it is actually a classic risk behavior that in accordance to the “problem behavior theory” belongs to the cluster of alcohol, tobacco, and drug use. The findings also showed that among the sample of Arab youth, strong relationships existed between poor communication with parents and negative school perceptions and water-pipe use (18). As the PBT suggests that there was a statistically significant correlation between cigarette and water-pipe smoking in populations where there is low-religious inclination, increased parental smoking, and low-student academic achievement.

Another HBSC study examined the roles of parents (monitoring, involvement, and support with school), teachers (support), and peers (excess time spent with friends, peer rejection at school) in predicting risk behaviors (smoking and drinking) and mental well-being. The study was conducted among 3499 Israeli-born and 434 Israeli immigrant adolescents aged 11, 13, and 15, in 2006 (19). Structural equation modeling (SEM) showed that for native Israeli youth all three relationships – parents, teachers, and peers – have a significant impact on both mental well-being and risk behaviors. However, for immigrant adolescents, it was the school environment that proved to be the most significant predictor of risk behaviors and mental health outcomes.

In this paper, we re-examine the applicability of the PBT on an additional young adult undergraduate population and explore the contribution of psychosocial protective and risk factors have on the explanation of problem behaviors among young adult students in a large academic institute in Israel. We seek answers to the question: are there are any protective factors that moderate, or buffer, the impact of risk factors in this sample and what social context may we find them in?

## Materials and Methods

### Questionnaire

This study was based on a structured, self-reported, anonymous questionnaire distributed to undergraduate students in a large academic institution in Israel. The origin of the majority of the questions was Jessor’s Survey of *Personal and Social Development*, University of Colorado (20), which was culturally adapted to the Israeli student population. The topics included in the questionnaire were socio-demographic parameters, self perception, health perceptions, self and body image, psycho-somatic questions, emotional stressors, social support, social relationships, academic field of study, religion, risk behaviors, nutrition, and physical activity. The questionnaire was distributed in two rows in order to shorten the completion time within the classrooms. Both rows contained core questions on each of the topics, but “burdening” questions were presented in only one of the two rows. Row 1 contained an expansion on risk behaviors such as smoking, alcohol, driving violation, and drugs. Row 2 contained an expansion on studying, religion, security, nutrition, and sexual intercourse.

### Participants

The sample included 1,360 undergraduate students (882 females and 478 males) studying in the faculties of Health Sciences, Natural Sciences, and Social Sciences in a large academic institution in Israel, between April and May 2009. The sample was comprised of approximately one-third of all registered undergraduate students at these faculties. The response rate was 93.5%; of this, a total of 686 students completed Row A of the questionnaire, 669 completed Row B of the questionnaire, and 5 students completed the pilot questionnaire. The mean age of the respondents was 25 (SD = 2.9, range = 17).

### Procedures

Ethics Committee approval was obtained from the academic institution prior to the pilot phase. The sample was a convenience sample-questionnaires were distributed in classes where the lecturers permitted so. Detailed guidance was provided to the research staff as to the technique of presenting the questionnaires within the classrooms, and they were also trained on research ethics. All students present in participating classes received a questionnaire to be completed inside the classroom 10 min before the end of the lesson. The research staff read an introduction before handing out the questionnaire and allowed students the opportunity of declining participation. Respondents who chose to complete the questionnaire were requested to give signed consent. In total, the survey team entered approximately 70 classrooms during April–May 2009.

### Data analysis

A quantitative analysis was conducted in this study by using SPSS software. The final file included students until the age of 34 and divorced or widowed students were removed from the sample. The first phase examined descriptive statistics of frequencies by age group and gender. Significance was checked for by the chi-square test to examine the independence between variables using the crosstabs column proportion test. In the second phase, a hierarchal linear regression was conducted for the dependent variable multiple problem behavior index (MPBI). And the third phase introduced an interaction graph between variables by a two-way analysis of variance (two-way ANOVA).

### Measures

The explained variables used to construct a MPBI of protective and risk factor measures in the model are described in Table 1. The measure is composed of 17 different risk behaviors on the topics: cigarette smoking, hookah smoking, risky driving, vandalism, violence, academic fraud, and risky sexual behavior.

**Table 1:** **Description of items used to construct a MPBI of protective and risk factor measures**.

Question	Response code
**Individual controls protective factors**
**Individual controls protective factors-religion (Cronbach’s alpha 4 items = 0.944)**
How important is it to you:
To be able to rely on religious teachings when you have a problem?	1. Not important at all
To believe in god or a higher power of creation?	2. Not too important
To rely on your religious beliefs as a guide for day-to-day living?	3. Somewhat important
To be able to turn to prayers when you are facing a personal problem?	4. Very important
**Individual controls protective factors-academic achievements (Cronbach’s alpha 4 items = 0.707)**
How important is it to you:
To get at least a B average this year?	1. Not important at all
To be considered a bright student by your teachers?	2. Not too important
To come out near the top of the class on exams?	3. Somewhat important
To have good enough grades to get into graduate or professional school if you would like?	4. Very important
**Opportunity risk factors**
If you would want some beer, wine or liquor, how hard would it be to get it?	1. Difficult
	2. Fairly easy
	3. Very easy
Are your parents living together?	1. Yes
	2. No
**Vulnerability risk factors (Cronbach’s alpha 6 items = 0.672)**
In the past month, how much stress or pressure have you felt due to?
Your schoolwork?	1. None at all
Where you live?	2. Only a little
Your family life?	3. A fair amount
Your personal or social life?	4. A lot
During the past 2 weeks, to what extent do you feel that your life is under threat?	1. Not at all
To what extent do you feel that the lives of your family members or of other people are at risk?	2. A little
	3. Average
	4. A lot
	5. Extremely
**Dependent variable-MBPI**
**Problem behavior involvement index-increasing values in the new variables reflects greater MPBI levels (Cronbach’s alpha 17 items = 0.628)**	
Have you ever smoked a cigarette (not just a few puffs)?	1. No, never
	2. Yes, but only once
	3. A few times
	4. More than a few times
Did you ever smoke a water-pipe (Hookah)?	1. Yes
Have you ever had a drink of beer, wine or liquor – not just a sip or a taste of someone else’s drink?	2. No
Have you ever tried Marijuana (or hash)?	1. No, never
	2. Yes, once,
	3. Yes, more than once
During the past month, how often did you:
Drive through a stop sign without coming to a full stop?	1. Never
Drive too close to the car in front of you (“tailgate”)?	2. Once or twice
Drive after you drank at least a whole beer, a glass of wine, or something like that?	3. Three–four times
Drive more than 20 kph over the speed limit?	4. Five–nine times
Drive through a red light?	5. 10 times or more
Drive after you had used marijuana?	
During the past month, how often did you:
Cheat on a test or homework?	1. Never
Shoplift from a shop?	2. Once
Damage or mark up public or private property on purpose?	3. Twice
Sell or deal drugs?	4. Three–four times
Steal something valuable, like someone’s Palm Pilot, backpack or wallet?	5. Five times or more
Hit someone because you did not like what he/she did or say?	
How old were you the first time you had sexual intercourse?	Age in years

Three socio-demographic variables were used in the hierarchal regressions: gender (male, female), marital status (single, married), and income (on a scale ranging from <7000 NIS per month to more than 25,000 NIS per month). The descriptions of all other variables are presented in Table 2.

**Table 2:** **Descriptive characteristics of study participants by gender and age-groups (%)**.

Characteristics	Up to 25 years old (*n* = 580)			26 years and older (*n* = 780)			Total (*N* = 1360)^a^
	Males (*n* = 174)	Females (*n* = 406)	Total younger	Males (*n* = 506)	Females (*n* = 274)	Total older	
**Ethnicity**
Ashkenazi (West Europe)	28.2	36.9	34.3	34.7	38.1	35.8	35.2
Mizrahi/Sephardic	38.2	44.4	42.6	42.2	39.4	41.3	41.8
ESSR/East Europe	17.3	12.4	13.9	19.5	16	18.3	16.4
Ethiopia	0	2.1	1.5	1.8	4.9	2.9	2.3
Other (Arab/Druze/Bedouin)	16.4	4.1	7.8	1.8	1.6	1.8	4.3
**Marital status**
Single	88.5	84	85.4	69.7	69.7	69.7	76.4
Married	11.5	16	14.6	30.3	30.3	30.3	23.6
**Have one or more children**	5.2	5	5.1	18.3	17	17.8	12.4
**Family monthly income**
Least	46.6	49.1	48.3	43.2	53.6	46.8	47.4
Middle	32	40.2	37.7	43.8	38.3	41.9	40.2
Most wealthy	21.4	10.7	14	12.9	8.1	11.3	12.4
**Permanent residence**
Ariel	10.8	9.4	9.8	13.8	14.6	14.1	12.3
Judea and Samaria (not Ariel)	19.2	22.7	21.7	18.9	7.3	14.8	17.7
Elsewhere in Israel	70	67.9	68.6	67.3	78.1	71.1	70
**Current residence**
Student dorms	19.2	24.9	23.2	14.3	13	13.9	17.8
Home, with my parents	60	50.1	53	35.8	30.8	34	42.2
In Ariel, outside of the campus, in a house	2.5	4	3.6	9.2	13.8	10.8	7.7
In a house or apartment at a different place	18.3	21	20.2	40.7	42.4	41.3	32.3
**Parental education**
Higher education	37	37.3	37.2	30.9	33.4	31.8	34.1
High-school education	63	62.7	62.8	69.1	66.6	68.2	65.9

*^a^All data were weighted by gender*.

## Results

Descriptive characteristics of the study participants by gender and age-groups are presented in Table 2. The majority of the students reported to be single (76.4%), and 12.4% reported having one or more children. Most of the students reported their family monthly income to be low (47.4%) or medium (40.2%). Only 17.8% of the student’s reside in the student dorms, and over 40% live at home with their parents. The majority of the students marked their ethnic origin as Mizrahi/Sephardic (41.8%) and Ashkenazi (Western Europe; 35.2%), and only a minority of the sample marked their ethnic origin as ESSR/Eastern Europe (16.4%), Ethiopian (2.3), or other (Arab/Druze/Bedouin; 4.3%). In accordance with the general Israeli population, the younger students (under 25) are also more frequently unmarried (85.4%) and have fewer children (5.1%) in comparison to older students (69.7 and 17.8%, respectively). Likewise, more of them reside in the student dorms (23.2%) or with their parents (53.0%) than the older students (age 26 and over; 13.9 and 34.0%, respectively).

Health risk behaviors of the study participants by gender and age-groups are presented in Table 3. In sum, a high level of substance use can be observed: the frequency of water-pipe experiencing (71.2%) is higher than the frequency of experiencing cigarette smoking (58.5%). More than one-quarter of the sample (27.2%) experienced illegal marijuana use.

**Table 3:** **Health risk behaviors of study participants by gender and age-groups (%)**.

Characteristics		Up to 25 years old (*n* = 580)			26 years and older (*n* = 780)			Total (*N* = 1360)^a^
		Males (*n* = 174)	Females (*n* = 406)	Total younger	Males (*n* = 506)	Females (*n* = 274)	Total older	
**HEALTH RISK BEHAVIORS (FOR MPBI)**								
1	Ever smoked cigarettes	61.5	42.8	48.3	66	66.6	66.2	58.5*
2	Ever smoked a water-pipe (Hookah)	75.4	59.4	64.2	78.5	72.3	76.4	71.2*
3	Ever drank alcohol	78.4	70.7	73	88	82.7	86.2	80.4*
4	Ever used marijuana	22.9	14.5	16.9	33.6	37.4	34.9	27.2*
5	Drove through a stop sign without a coming to a full stop	45	40.7	42.2	38.8	25.6	34.7	37.7*
6	Drove too close to the car in front of you (“tailgate”)	66.7	55.9	59.6	62.2	54.8	59.8	59.7
7	Drove after drinking at least a whole beer, a glass of wine, or something like that	47	17.7	27.8	39	26.1	35	32.0*
8	Drove more than 20 kph over the speed limit	77.8	67.4	71	80.4	66.9	76.1	74
9	Drove through a red light	15.8	6.6	9.8	9.1	7.4	8.6	9.1
10	Drove after using marijuana	12	2.6	5.8	11.8	4.5	9.6	8.0*
11	Cheated on a tests or homework	31.7	26.2	27.8	25.7	22.4	24.6	26
12	Shoplifted from a shop	9.9	1.7	4	3.6	3.3	3.5	3.7
13	Damaged or marked up public or private property on purpose	13.9	2.4	5.6	4.9	1.3	3.6	4.5
14	Sold or dealt drugs	8	1.7	3.5	3.6	4	3.7	3.6
15	Stole something valuable like a palm pilot computer, backpack or wallet	6.9	1.1	2.7	1.3	0.3	1	1.7*
16	Hit someone because you did not like what he/she did or said	10.9	3	5.2	5.2	2	4.1	4.6
17	Had sex before the age of 15	19.3	4.1	9	15.2	4.3	11.3	10.5

*^a^All data were weighted by gender*.

*p < 0.05 – *z* test to check column proportion to significant differences between younger and older groups.

Differentiation can be seen between age-groups and gender in relation to health risk behaviors. In the younger population (up to 25 years old), the frequency of all 17 risk behaviors is higher among males than among females. For example, major gaps were found in experiencing cigarette smoking (61.5 vs. 42.8%), sex prior to the age of 15 (19.3 vs. 4.1%), driving after drinking (47.0 vs. 17.7%), or driving after using marijuana (12.0 vs. 2.6%). It can be observed that also among the group of older students, the prevalence of risk behaviors, almost in all instances, is higher among the males.

About 10.5% of the students in the sample reported that they had sexual intercourse before the age of 15, with a higher prevalence among males in both age groups. All risky driving variables show a higher prevalence among males than among females in both age groups. The frequency of speeding (more than 20 km/h over the speed limit) was reported by the majority of the sample and stands at 74.0%. Cheating on exams or homework was reported by about a quarter of the sample (26.0%) with a slightly higher incidence among the younger students (27.8%) than the older ones (24.6%). It can be observed from the data that in sum, substance experimentation is higher among the older students than among the younger ones.

Table 4 presents the hierarchal linear regression of the MPBI on socio-demographics, protective, and risk factors among the students. The sample was weighted by gender. The MPBI was built of 17 risk behaviors.

**Table 4:** **Hierarchal linear regression of the multiple problem behavior index^b^ on socio-demographics protective factors, and risk factors among students^a^**.

		I	II	III	IV	V	VI
Socio-demographic factors	Gender (0** = **female, 1** = **male)	0.202***			0.227***	0.211***	0.239***
	Marital status (single** = **1; married** = **0)	0.074*			0.086*	0.070*	0.086*
	Income (0** ≤ **7,000 NIS, 6** = ** more than 25,000 NIS)	0.110***			0.120**	0.119***	0.121**
Protective factors	Religious beliefs (0** = **less religious … 4** = **more religious)		−0.111**		−0.107*		−0.111**
	Individual controls protective factors academic achievements (including items study 1–4; 0** = **less … 4** = **more)		−0.107**		−0.086*		−0.096*
Risk factors	Opportunity risk factor (acquiring alcohol)			0.149*		–	–
	Parents live together (0** = **yes, 1** = **no)			−0.150*		–	–
	Vulnerability Risk Factors (self + security)			0.253***		0.102***	0.098*
	Adjusted *R*^2^	0.062***	0.022***	0.092***	0.097***	0.071***	0.104***
	*N*	1,150	626	234	534	1,150	534

^a^Weighted sample by gender. Significant: *p < 0.05, **p < 0.01, ***p < 0.001.

*^b^The multiple problem behavior index (MPBI) was built of 17 risk behaviors shown in the continuation of Table 1*.

*All behaviors were standardized and the mean was computed*.

The final regression model was built in stages as detailed: in the first stage, the socio – demographic variables were added to the model. In the second stage, the protective variables were added. In the third stage risk variables were added. In the fourth stage, both the socio-demographic variables and protective variables were added together. In the fifth stage, both socio-demographic variables and risk variables were added together. In the sixth and final stage, all distinct variables from all of the previous stages and all three variable groups were entered into the model.

All behaviors were standardized and their mean was computed. As can be seen, in all of the regressions, gender, marital status, and income influence risk behaviors. Religiosity was found to be a strong and significant protective factor which lessens risk behaviors. In addition, high-academic achievements also contribute to reducing involvement in risk behaviors, while vulnerability risk factors (family pressure and the national security situation) increase the involvement in risk behaviors.

The findings indicate that, in fact, chances of being involved in risk behaviors are higher if you are male, single, secular, with a high income, lower academic aspirations, and feel stress in your personal life.

In the final regression model, the two additional risk factors (opportunity and parents living together) were not found to be significant. Likewise, the variables of age, parenthood, or parental education were not significant in effect in the final model. The final regression model explains about 10% of the variance.

**Figure 1:**
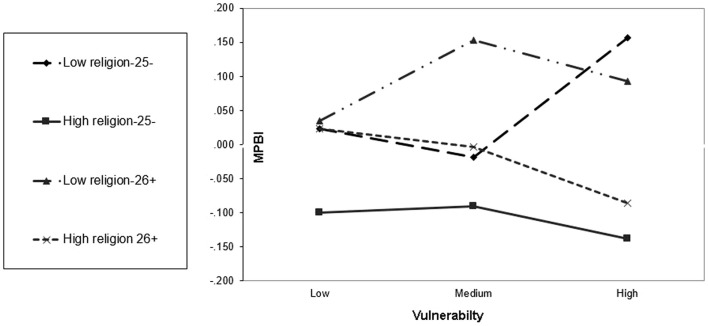
**Moderating effects of the protection factors (religion) and hazardous effects of the risk factor (vulnerability) on the MPBI among younger (25 or less) and older (26+) students**. Religiosity values: combinations of 4 questions 0–3 = less religious, 4 = more religious. Younger group *n* = 294, older group *n* = 317.

Figure 1 demonstrates the interaction between the religiosity and vulnerability risk factors and their affect on the dependent composed MPBI variable. A moderating effect of religiosity on several risk behaviors can be observed both among the younger students, as well as among the older students. For the younger group, vulnerability was found to have nearly no effect when religious levels are high; while the involvement in risk behaviors clearly raises when the vulnerability is higher among those with low levels of religiosity. For the older group, the MPBI was found to be higher when religiosity levels were lower, but when the vulnerability was low, there is no clear difference between the high- and low-religiosity levels on the MPBI.

Among the older group, when the vulnerability is low, the religiosity has almost no effect on involvement in risk behaviors. Yet, as the vulnerability increases, the differences between religious and non-religious individuals are more substantial. As found to be true for the younger group, high-religious involvement reduces the risk of involvement in risk behaviors.

Among secular individuals, the impact of the vulnerability varies between the younger and older groups. While risk behaviors among the younger group rise significantly when vulnerability increases, among the older group there is moderation in the rise of involvement in risk behaviors when vulnerability increases. Thus, when people are young and religious, the personal vulnerability has almost no impact on involvement in risk behaviors, and the influence of religion is strong. In other words, religion is a protective factor against personal vulnerability.

## Discussion

The current study tested the applicability of the PBT among undergraduate students in a large academic institution in Israel. In this sample, the participants were young adult undergraduate university students, mean age 25. The PBT explains that behaviors such as delinquency, tobacco use, alcohol abuse, other illicit drug use, early sexual intercourse, aggression, and risky driving, are a product of integration between risk factors and the moderating or buffering effects of protection factors, which may have an impact on exposure to risk. As the literature shows, the PBT had been examined before in various samples of young adolescents in the US, China, Georgia, Switzerland, and sub-Saharan Africa. The uniqueness in our study, beside the fact that it has never been tested in Israel before, is that the participants were all young adults or at their last stage of their adolescence, with mean age 25 (SD = 2.9, range = 23), the majority of them registered to study after serving 2–3 years in the military, about 23% of them were already married and 11.6% are parents. So, the question that this study raises is whether the PBT is applicable for this age group and for these additional characteristics.

An additional important variant differentiating between the supporting studies is that the risk behaviors, the risk factors, and the resilience factors in each of the studies are different. In this article, the risk behaviors refer to substance use, including hookah smoking as a unique phenomenon, risky driving, cheating, and delinquent behaviors and risky sexual behavior. The results in this study exhibit 17 different risk behaviors, which participants have reported to be involved in, as reported in the literature, all were found to be more frequent among males than among females.

The findings indicate that a large sample of students who had experienced various substances. The frequency of experiencing cigarette smoking was more than half of the sample, and the frequency of experiencing hookah smoking was over 70% of the students. Over a quarter of the sample reported illegal marijuana use. The PBT demonstrates that risk behaviors of substance use have a clustering effect – strong relationships with other risk behaviors (6), and other studies suggest that the association between hookah smoking, cigarette smoking, problematic alcohol consumption, and drug use is very strong (16, 17).

In addition to the risk behaviors of substance use, another common behavior is risky driving, which characterizes the sample: the frequency of speeding (over 20 km/h over the speed limit) was reported by the majority of the sample and stands at 74.0%. Similarly, about a quarter of the sample reported cheating on exams or homework at least once within the past month. This article attempts to find the determinants of these risk behaviors.

The regression model was used to identify the factors that constitute resilience and risk from the MPBI. Significant resilience factors found were religion (strength of the religious faith) and academic ambitions (the importance of grades and the appreciation for the individual as a student). As there was found in the literature, significant correlation between cigarette and water-pipe smoking in populations where there is low-religious inclination, increased parental smoking, and low-student academic achievement (18). Significant risk factors found were personal vulnerability (feelings of stress in life and the sense of danger due to a security threat). The findings show that, in fact, chances of being involved in risk behaviors are higher if you are male, single, secular, with a high income, lower academic aspirations, and feel stress in your personal life.

The examination of the interactions of protective and risk factors on the MPBI demonstrated that the strong religious beliefs a person has protect him from personal vulnerability, and that in the older age group, the age must play a part as vulnerability is less meaningful. Our study findings indicate that the PBT was indeed replicated in our sample. The present study showed that protective and risk factors play a significant role in the various problematic risk behaviors exhibited by this sample of undergraduate students.

The findings of this study are consistent with the literature, persuasive, and to some extent, not surprising. Logically, and in similar to what occurs in other countries around the world, and within other age groups, we also observed similar trends. That is, even though the cultural context is different, the etiology still remains.

The findings of this study have different various educational and social implications. Within the academic framework, problematic behaviors may be reduced by identifying risk groups prone to these problematic behaviors. Various programs targeted at reducing personal feelings of pressure and stressors, resulting from various reasons, within the home, the family, the educational system, and the national security environments. It may be that the risk behaviors are a form of maladaptive coping behaviors with stressors and risk factors in the personal and security environment in the already stressful life situation of the Israeli student. It is clear that not all risk factors may be treated, and that it is not always possible to strengthen protective factors, but the understanding that they present risk and protective factors is significant. Intervention strategies should set a goal of reducing both exposure to problematic behaviors and their adoption, and the thorough treatment of risk factors while strengthening resilience factors.

One of the limitations of this study is its cross-sectional nature. A longitudinal study could have yield sharper results within the causal context. Another limitation of the study is that it used the method of self-report, which may lead to bias based on social desirability. Future research should conduct similar studies among undergraduate students while taking into account the points made above.

## Conflict of Interest Statement

The authors declare that the research was conducted in the absence of any commercial or financial relationships that could be construed as a potential conflict of interest.
